# Transcatheter patent ductus arteriosus closure in very low birth weight preterm infants: early results and midterm follow-up

**DOI:** 10.3389/fped.2025.1650335

**Published:** 2025-07-28

**Authors:** Junhui Liu, Wei Gao, Zigang Liu, Kun Zhao, Gang Luo, Shuai Gao, Yi Sun, Silin Pan

**Affiliations:** ^1^ Heart Center, Women and Children’s Hospital, Qingdao University, Qingdao, China; ^2^ Cardiology, Shanghai Children’s Medical Center, School of Medicine, Shanghai Jiao Tong University, Shanghai, China; ^3^ Cardiothoracic Surgery, Baoan District Maternal and Child Health Hospital, Shenzhen, China; ^4^ Heart Center, Northwest Women’s and Children’s Hospital, Xian, China

**Keywords:** transcatheter closure, patent ductus arteriosus, very low birth weight, preterm infants, midterm follow-up

## Abstract

**Background:**

Although transcatheter patent ductus arteriosus (PDA) closure is becoming increasingly common in very low birth weight (VLBW) preterm infants, several key issues remain controversial. These include identifying suitable patient characteristics, determining the optimal timing for PDA closure, preventing potential complications, and accurately assessing mid- and long-term outcomes. This study aims to summarize our preliminary experience in selecting appropriate patients and timing for PDA closure, and to report the early and mid-term outcomes of transcatheter PDA closure in VLBW preterm infants.

**Methods:**

This was a single-center retrospective study. Eligible participants included preterm infants with gestational age <37 weeks and birth weight <1,500 g who underwent transcatheter PDA closure between January 2024 and January 2025 at Qingdao Women and Children's Hospital. Data on patient characteristics, procedural age, PDA closure, survival, and intraoperative or postoperative complications were collected. Outcomes were assessed immediately after the procedure, at discharge, and 6 months post-discharge.

**Results:**

Procedures were performed in 8 VLBW preterm infants [median procedural age 23 days (range: 13–36 days), median procedural weight 1,350 g (range: 810–1,480 g), median PDA diameter 3.75 mm (range: 2.3–4.1 mm)]. The devices were Amplatzer Piccolo (*n* = 8). Procedures were successful in 100% and uneventful in 87.5% (7 of 8). One patient experienced mild left pulmonary artery compression intraoperatively, which resolved with device repositioning. 25% (2 of 8) patients experienced transient systemic hypertension within 24 h postoperatively, which resolved with diuretic and sedative treatment. No patients experienced ventilation or oxygenation failure, residual PDA, device malposition, or embolization. Survival to discharge was 100%. At 6-month follow-up, all patients were alive and well, without residual PDA, left pulmonary artery stenosis, and aortic coarctation.

**Conclusions:**

The promising early and mid-term outcomes suggest that transcatheter PDA closure in VLBW preterm infants is feasible. Suitable patient characteristics, accurate PDA closure timing, and careful postoperative care are crucial determinants for procedural success. Future studies need to further expand the sample size and extend the follow-up period to evaluate the long-term efficacy and potential complications of this intervention.

## Introduction

Patent ductus arteriosus (PDA) is very common among preterm infants, with its incidence closely linked to gestational age and birth weight. The spontaneous closure rate of ductus arteriosus (DA) in full-term newborns is as high as 99.95% within 72 h after delivery. However, most preterm infants experience delayed closure. As gestational age and birth weight decrease, particularly in very low birth weight (VLBW) preterm infants, the incidence of PDA significantly rises (1). However, whether to close, how to close, and when to close the PDA remain controversial.

There is a lack of high-quality evidence indicating that PDA closure is beneficial for outcomes of VLBW infants. Mitra et al. recommend that definitive PDA closure should be considered only in populations at high risk of death or with severe bronchopulmonary dysplasia (BPD) (2). Krishnappa et al. suggested that infants with a large persistent PDA shunt are more likely to benefit from PDA closure (3). Non-interventional management may benefit certain infants, but some infants still require further intervention (4, 5). Pharmacotherapy fails in approximately 50% of extremely preterm infants (6) and leads to renal and gastrointestinal complications in some cohorts (7). Some studies indicate that surgical ligation is not superior to transcatheter PDA closure (8, 9). Numerous studies (10–12) have shown positive outcomes of transcatheter PDA closure in increasingly younger infants. Mitra et al. emphasized that, with adequate institutional expertise and appropriate patient characteristics, transcatheter PDA closure could be prioritized over surgical ligation.

The timing of PDA closure is critical for infant outcomes. Many studies highlighted that the persistent PDA shunts increased the risks of BPD, chronic lung disease, and mortality (13–15). A multicenter study demonstrated that preterm infants with moderate-to-large PDA, who require prolonged tracheal ventilation for ≥10 days, are at increased risk of BPD and death (16). Shi et al. pointed out that most VLBW infants with high-flow PDA may become ventilator-dependent and develop BPD and pulmonary hypertension by 6 weeks of age (17).

This study aims to summarize our preliminary experience in selecting appropriate patients and timing for PDA closure and to report the early and mid-term outcomes of transcatheter PDA closure in VLBW preterm infants.

## Materials and methods

### Study participants

This study is a retrospective study, and the participants are VLBW preterm infants who underwent transcatheter PDA closure at Qingdao Women and Children's Hospital between January 2024 and January 2025. In this study, transcatheter PDA closure was indicated in VLBW preterm infants with hemodynamically significant PDA (hsPDA) who failed to close the PDA with conservative and pharmacologic therapy and had at least one of the following clinical symptoms: congestive heart failure, growth retardation, increased pulmonary blood flow, left atrial or ventricular enlargement, or ventilation dependence. The diagnostic criteria of hsPDA (18): There is continuous left-to-right blood flow within the duct, with the ductal diameter ≥1.5 mm or the ratio of duct-to-left pulmonary artery diameter ≥0.5, along with at least one of the following criteria: (1) the ratio of left atrium-to-aortic root diameter ≥1.5; (2) the velocity of ductal blood flow ≤2.5 m/s or the average pressure difference across two ends of duct ≤8 mmHg; (3) presence of diastolic blood flow reversal in the descending aorta. Exclusion criteria: severe congenital anomalies, complex congenital heart defects, and hereditary metabolic diseases. The study was approved by the Institutional Review Board of Qingdao Women and Children's Hospital (QFELL-YJ-2024-180).

### Procedure details

**Figure 1:**
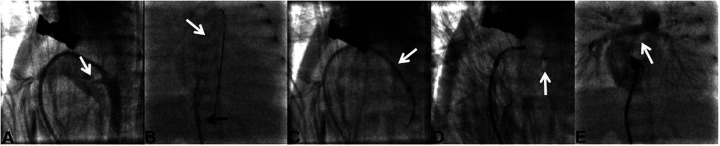
Procedure details in case 1. **(A)** In case 1, the PDA (white arrow) diameter was 2.3 mm and the PDA type was type C. **(B)** Delivery sheath (black arrow) advanced over a 0.014-inch guidewire (white arrow). **(C)** Delivery sheath (white arrow) was positioned across the PDA. **(D)** Amplatzer Piccolo occluder (white arrow) was released. **(E)** Pulmonary arteries (white arrow) without stenosis were detected by angiography.

The procedure was performed under general anesthesia. Preoperative ultrasound measured the diameter of the right femoral vein, followed by ultrasound-guided puncture and insertion of a 4-Fr sheath. A 4-Fr Vertebral catheter (RF*WH14110M, Terumo Corporation, Japan) was advanced via the femoral vein, inferior vena cava, right atrium, right ventricle, pulmonary artery, and DA into the descending aorta. Angiography was performed by hand-injecting contrast medium to confirm the length, shape, and diameter of the PDA. A 0.014-inch BMW guidewire was inserted to establish a delivery track, and the Vertebral catheter was removed. An appropriately sized Amplatzer Piccolo Occluder (Abbott, USA) was selected. The selected occluder waist diameter should be at least 1 mm larger than the measured ductal diameter to prevent device migration. The occluder length should be equal to or shorter than the measured ductal length to avoid stenosis of adjacent vessels. The Amplatzer TorqVue LP catheter (Abbott, USA) was advanced over the guidewire into the descending aorta. Then the guidewire and inner core were removed. The occluder was implanted within the DA. The aortic disc was released first, and angiography confirmed proper positioning of occluder. Echocardiography verified no residual shunt and no stenosis in the pulmonary artery or aorta. The pulmonary disc was then released to complete occlusion. After the procedure, the catheter was removed. Details of the procedure are shown in Figure 1.

### Procedural data and follow-up

Data were collected from Qingdao Women and Children's Hospital. Data include gestational age (weeks + days), sex, birth weight (g), procedure age (day), procedure weight (g), PDA diameter (mm), PDA type, procedural time (min), device, preoperative blood pressure (BP) (mm/Hg), postoperative BP (mm/Hg), PDA closure, procedural complications, heart rate (HR), oxygen saturation (SpO_2_). Systemic hypotension is defined by a systolic BP less than the third percentile for postmenstrual age (PMA) (19) during any of the timepoints. Systemic hypertension is defined by systolic BP greater than the 95th percentile for PMA (20) during any of the timepoints or using new inotropes/vasopressors in the first 24 h after catheterization. Ventilation failure is defined as a need to escalate to high-frequency ventilation when conventional ventilation strategies fail within 24 h of catheterization (19, 21). Oxygenation failure is defined as an absolute increase of ≥20% in the fraction of inspired oxygen or mean airway pressure compared with the immediate post-catheterization value within 24 h of catheterization (19).

Efficacy evaluation: (1) Accurate placement of the occluder, confirmed by angiography or echocardiography. (2) Successful PDA closure. (3) Improvement of related clinical symptoms, such as congestive heart failure, left atrial or ventricular enlargement, pulmonary hemorrhage, necrotizing enterocolitis, intraventricular hemorrhage, and growth and development, successful weaning from mechanical ventilation, maintenance of vital signs, such as SpO_2_, BP, and HR, within normal ranges, and no occurrence of new PDA-related complications.

Safety evaluation: (1) Severe procedural complications include pericardial tamponade, cardiac perforation, device malposition, pulmonary artery stenosis, aortic coarctation, etc. (2) Mild procedural complications include puncture site hematoma, transient arrhythmia, and transient blood pressure fluctuation. The study endpoints are set as the PDA closure rate and procedural complication rate at 6 months after procedure.

## Results

The study enrolled 8 VLBW preterm infants, including 6 males and 2 females. The gestational ages ranged from 24 to 29 weeks, and the median birth weight was 1,025 g [range: 670–1,280 g]. The median PDA diameter was 3.75 mm [range: 2.3–4.1 mm], and the median PDA length was 8.15 mm [range: 7.1–11.2 mm]. The most common type of PDA was type F (fetal type) (*n* = 6). The median age and weight at the time of the procedure were 23 days [range: 13–36 days] and 1,350 g [range: 810–1,480 g]. Cases 1 and 5 received two cycles of ibuprofen (10 mg/kg, 5 mg/kg, and 5 mg/kg at 24-h intervals), while the remaining cases received one cycle of ibuprofen. Pharmacotherapy to close the PDA failed in all patients. Demographic and clinical characteristics of the patients are shown in Table 1.

**Table 1:** Demographic and clinical characteristics of patients.

Case	Sex	Gestational age (weeks + days)	Birth weight (g)	Procedure age (days)	Procedure weight (g)	PDA diameter (mm)	PDA length (mm)	PDA type
1	Male	24 + 2	670	33	810	2.3	7.6	C
2	Male	29 + 4	1,000	13	1,000	3.8	7.2	F
3	Female	25 + 3	830	25	1,200	4.1	11.2	F
4	Female	29 + 2	1,220	23	1,300	4.1	10.4	F
5	Male	28 + 1	1,050	36	1,400	3.7	9.2	F
6	Male	28 + 6	980	21	1,430	3.6	8.7	F
7	Male	29 + 6	1,280	20	1,450	4.1	7.1	C
8	Male	29 + 5	1,210	23	1,480	3.1	7.5	F

Femoral vein puncture was successfully completed in all 8 patients with a 100% success rate, and no puncture-related complications occurred. The median procedural time was 48 min [range: 38–98 min]. Devices used in the procedures were Amplatzer Piccolo (*n* = 8). Procedures were successful in 8 patients (100%) and uneventful in 7 patients (87.5%). One patient experienced transient left pulmonary artery (LPA) compression intraoperatively. The overall rate of systolic hypertension was 25% (2 of 8) postoperatively. None of the patients developed systolic hypotension, oxygenation, and ventilation failure. There were no procedure-related deaths, residual shunt, or device malposition and embolization. The median postoperative mechanical ventilation time was 8.5 days [range: 3–32 days]. Procedural data and complications are summarized in Table 2.

**Table 2:** Procedural data and outcomes.

Case	Procedural time (min)	Device	Device size (mm)	PDA closure	Preoperative BP (mm/Hg)	Postoperative BP (mm/Hg)	Postoperative mechanical ventilation time (days)	Complications
1	98	Amplatzer Piccolo	4 × 4	Closed	59/32	72/39	32	Transient LPA compression
2	40	Amplatzer Piccolo	5 × 4	Closed	46/25	55/31	7	–
3	75	Amplatzer Piccolo	5 × 4	Closed	47/32	59/39	28	–
4	55	Amplatzer Piccolo	5 × 4	Closed	55/29	65/35	11	–
5	43	Amplatzer Piccolo	5 × 4	Closed	85/45	94/60	3	Transient systolic hypertension
6	53	Amplatzer Piccolo	5 × 4	Closed	51/28	67/36	10	–
7	38	Amplatzer Piccolo	5 × 4	Closed	83/41	95/61	7	Transient systolic hypertension
8	42	Amplatzer Piccolo	5 × 4	Closed	56/33	71/48	3	–

At discharge, the survival rate was 100%. Severe complications were not observed in 8 patients. The SpO_2_ of all patients remained stable at 91%–95%, BP at 50–80/30–50 mmHg, and HR at 120–140 bpm. At the 6-month follow-up, 8 patients were alive and well, and maintained normal levels of SpO_2_, BP, and HR. Residual shunts, LPA stenosis, aortic coarctation, device malposition, and embolization were not observed in all patients.

## Discussion

Although transcatheter PDA closure has become the standard intervention in most cases (2), several controversies remain, such as suitable patient characteristics, the optimal timing of shunt elimination, the prevention of complications, and the assessment of long-term prognosis. The absence of an evidence-based management strategy regarding transcatheter PDA closure in VLBW infants motivated our group to investigate the optimal strategy for performing this procedure within this population. Our study reported a 100% success rate of PDA closure, without major intraoperative or postoperative complications. This aligns with previous studies, where Morville et al. (22) and Sathanandam et al. (23) reported success rates of 94% and 98%, respectively. Ventilators were successfully weaned off after the procedure, and the survival rate at discharge was 100%. At a follow-up period of 6 months, all patients were alive and well, without severe complications. These findings support the feasibility of transcatheter PDA closure in VLBW preterm infants.

For VLBW preterm infants with failed pharmacotherapy, interventions for PDA closure include surgical ligation and transcatheter closure. Compared with transcatheter closure, surgical ligation is associated with higher risks of infection and bleeding, as well as longer recovery and hospitalization durations (2). However, studies have shown no significant difference in PDA closure success rates between the two approaches, with the incidence and type of adverse events primarily linked to the expertise and experience of the medical team (8, 9). In this study, 8 VLBW preterm infants showed persistent PDA despite conservative management and at least three doses of ibuprofen, and developed complications such as prolonged ventilation, feeding intolerance, and pulmonary edema, indicating the need for further intervention. Considering the patients' multiple comorbidities, poor tolerance, and the trauma of surgical ligation, along with our center's extensive experience in transcatheter closure, we ultimately selected transcatheter closure for all 8 cases.

Clearly defining the patient characteristics suitable for transcatheter PDA closure is crucial. Mitra et al. (2) proposed that PDA closure should be considered only in infants at high risk of mortality or development of BPD. A single-center retrospective study of VLBW preterm infants found that the risk of death in preterm infants with persistent PDA shunts was eight times higher than that in infants with closed PDAs (15). Krishnappa et al. found that large PDA diameter (>2.5 mm) and left ventricular dilatation (*z* score ≥2) were associated with earlier extubation after PDA closure (3). Clyman et al. (16) suggested that PDA closure is necessary for VLBW preterm infants with moderate-to-large hsPDA who require tracheal ventilation for more than 10 days, as this increases the risk of death and BPD. Mitra et al. suggested that a second course of pharmacotherapy should be attempted before procedural PDA closure, unless there are contraindications to medications in infants (2). Therefore, we believe that transcatheter PDA closure may be considered for VLBW preterm infants with moderate-to-large hsPDA who require continuous mechanical ventilation for more than 10 days and have either failed a second course of pharmacotherapy or have contraindications to medications. Based on these criteria, the VLBW preterm infants in our study were considered for transcatheter PDA closure and achieved satisfactory short- and mid-term follow-up results.

Moreover, the timing of PDA closure may impact the outcomes of infants. Many studies highlighted that the persistent PDA shunts increased the risks of BPD, chronic lung disease, and mortality (13–15). VLBW infants undergoing transcatheter PDA closure within the first 4 weeks after birth may obtain more benefits, such as the prevention of early pulmonary vascular diseases, promotion of growth and development, improvement of respiratory function, and reduction of the ventilator and oxygen support (24, 25). Most VLBW infants with high-flow PDA may become ventilator-dependent and exhibit signs of BPD and pulmonary hypertension by 6 weeks of age (17). Therefore, we believe that the procedural age from 10 days to 6 weeks after birth may be the optimal timing of transcatheter PDA closure in VLBW preterm infants. In the present study, we performed the procedure in infants with a procedural age ranging from 13 to 36 days. There was no significant difference in the short and mid-term outcomes between the infant with an earlier procedural age (13 days) and the infant with a later procedural age (36 days).

The prevention of complications was another critical factor influencing infants' outcomes. In this study, while no severe procedural complications occurred, 1 infant experienced transient LPA compression during the procedure, which resolved after adjusting the occluder position. Additionally, 2 infants developed transient systemic hypertension after the procedure, which was managed with diuretics and sedation. This is consistent with the findings of Adrianne et al. (19), who reported that 43.6% of preterm infants developed systemic hypertension after this procedure. Persistent systemic hypertension is associated with secondary pulmonary venous hypertension and pulmonary edema, impairing respiratory function (19). Therefore, continuous blood pressure monitoring after the procedure is essential for the timely management of hemodynamic instability. Previous studies have reported procedural complication rates of 4.7%, 8.7%, 5.9% and 3.6% (9, 11, 22, 23), including pericardial effusion, LPA stenosis, aortic coarctation, tricuspid regurgitation, etc.

Our study reported a 100% survival rate at discharge. After a 6-month follow-up, all patients were alive without LPA stenosis, aortic coarctation, device displacement, residual PDA, or other severe complications. Narin et al. (10) followed up 19 preterm infants for 6 months after the procedure. 2 infants died from diseases unrelated to the procedure, and 4 infants developed mild LPA stenosis, resolved spontaneously during the 6-month follow-up. Zahn et al. (11) found that the survival rate of extremely preterm infants at discharge was 96%, and after a median follow-up period of 11.1 months, all patients were alive and well, without major complications. A prospective multicenter study followed up 200 infants weighing ≥700 g for 3 years after the procedure and reported that the implantation success rate was 95.5%, the PDA closure rate at 3 years was 100%, the overall survival rate was >95%, and 9 deaths occurred, all unrelated to the device or procedure (12). These results highlight the safety and efficacy of transcatheter PDA closure as a valuable treatment option for preterm infants with PDA.

In conclusion, this study confirms the feasibility of transcatheter PDA closure in VLBW preterm infants. However, the procedure is not suitable for all PDA cases. When selecting an intervention to close a PDA, it is essential to consider the technical expertise of the medical institution, the specific conditions of each patient, and the potential adverse effects of different interventions. Precise patient selection, optimal closure timing, and intensive postoperative care are critical for successful transcatheter PDA closure. Given that this was a single-center study with a small sample size and short follow-up period, future large-scale multicenter prospective studies with extended follow-up are needed to fully assess the long-term efficacy and safety of this intervention. Particular attention should be given to its impact on long-term quality of life, cardiac function, and neurodevelopment. This will provide solid evidence for its widespread clinical application.

## Data Availability

The original contributions presented in the study are included in the article/Supplementary Material, further inquiries can be directed to the corresponding author.
